# Features of Cryptic Promoters and Their Varied Reliance on Bromodomain-Containing Factors

**DOI:** 10.1371/journal.pone.0012927

**Published:** 2010-09-23

**Authors:** Samantha G. Pattenden, Madelaine M. Gogol, Jerry L. Workman

**Affiliations:** Stowers Institute for Medical Research, Kansas City, Missouri, United States of America; Texas A&M University, United States of America

## Abstract

The Set2-Rpd3S pathway is important for the control of transcription memory. Mutation of components of this pathway results in cryptic transcription initiation within the coding region of approximately 30% of yeast genes. Specifically, deletion of the Set2 histone methyltransferase or Rco1, a component of the Rpd3S histone deacetylase complex leads to hyperacetylation of certain open reading frames (ORFs). We used this mutant as a system to study the role of histone modifications and co-activator recruitment in preinitiation complex (PIC) formation. Specifically, we looked at the dependence of promoters on the bromodomain-containing RSC complex and the Bdf1 protein. We found that the dependence of cryptic promoters for these proteins varied. Overall, our data indicate that cryptic promoters are independently regulated, and their activation is dependent on factors that govern gene activation at canonical promoters.

## Introduction

Transcription by RNA polymerase II (Pol II) is a complex process that requires co-ordination of many factors, including chromatin modifying and remodeling enzymes, in order to elongate through the nucleosome barrier (reviewed in [1], [2], [3], [4]). Following transcription elongation, nucleosome deacetylation is important to prevent spurious transcription initiation within the open reading frame (ORF) [5], [6], [7]. The Set2-Rpd3S pathway mediates this process. The Set2 histone methyltransferase is associated with elongating Pol II [8], [9], [10]. It is responsible for the deposition of histone H3 lysine 36 trimethylation (H3 K36me3), a histone mark then recognized by the Rpd3S histone deacetylase complex, which subsequently erases histone acetylation in the ORF. This system is important for maintenance of genome integrity since mutations in components of the Set2-Rpd3S pathway lead to hyperacetylation and the production of cryptic transcripts within the ORFs of approximately 30% of yeast genes [11], [12].

While it is well known that mutation of components of the Set2-Rpd3S pathway produce cryptic transcripts, co-activators that affect transcription from cryptic promoters have not been well characterized. Cryptic transcripts initiate from the same position within the gene, but their levels vary depending on the mutant strain or growth conditions [13], which suggests that there are multiple mechanisms involved in cryptic promoter initiation. It remains largely unknown, however, if transcription is initiated from cryptic promoters in a manner similar to transcription initiation from the full length, or canonical gene promoter.

Bromodomain-containing proteins interact with acetylated histone tails and therefore are associated with initiation of transcription from active, acetylated promoters (Reviewed in [1], [2], [14]). Since the ORFs of genes that produce cryptic transcripts contain hyperacetylated histones [5], [6], [7], [11], bromodomain-containing proteins may be required for early transcription initiation from cryptic promoters.

Remodels Structure of Chromatin (RSC), is an essential ATP-dependent chromatin remodeling complex [15], [16], [17] that plays an important role in cellular processes such as chromosomal segregation, DNA repair, and transcription activation [18], [19], [20], [21], [22], [23], [24], [25], [26], [27], [28]. Subunits of the *Saccharomyces cerevisiae* RSC complex contain multiple bromodomains, which recognize acetyl lysine residues on histones and other proteins. For instance, the N-terminus of the Rsc4 subunit contains tandem bromodomains, one of which participates in binding acetylated Histone H3 lysine 14 (H3 K14Ac) [24]. RSC activity has been implicated in nucleosome repositioning and maintenance of the nucleosome free region (NFR) at RNA polymerase II (Pol II)-transcribed promoters [27], [28]. *In vitro*, purified RSC complex was shown to stimulate Pol II transcription through a nucleosome template; an event that was enhanced by NuA4 or SAGA-mediated histone acetylation [29]. Since RSC activity is linked to both nucleosome repositioning at promoters and the passage of Pol II through chromatin during transcription elongation, the RSC complex was a good candidate co-transcriptional activator for cryptic promoters.

Another bromodomain-containing protein that is important for the early recruitment of general transcription factors is Bdf1. This tandem bromodomain-containing protein has been shown to interact with the TFIID general transcription factor [30], [31], [32], the SWR1 complex, which is responsible for deposition of the H2A.Z histone variant [33], [34], [35], and with acetylated histone tails [36], [37], [38], [39], [40]. In *S. cerevisiae*, Taf1 does not have bromodomains; rather it pairs with Bdf1 as part of the TFIID complex [30], [31], [32]. Bdf1 is important for TFIID recruitment to about 90% of yeast genes [41]. These are mainly housekeeping genes that tend to be SAGA-independent and have TATA-less promoters [42], [43]. Due to its role in early transcription with the recruitment of TFIID to acetylated histones, Bdf1 is also an excellent candidate for regulation of cryptic promoters.

To investigate the role of the Rsc and Bdf1 bromodomain-containing proteins in activation of cryptic internal transcription, we exploited a mutant of the Rpd3S histone deacetylase complex, *rco1*Δ, which has an ORF hyperacetylation phenotype. This mutant was ideal for our studies because it could be used to determine the affect of histone acetylation on recruitment and activity of bromodomain-containing proteins and the role that these proteins play in cryptic promoter-formation.

## Results

### Mutation of the Rpd3S complex results in the appearance of promoter-associated histone modifications across the *STE11* gene ORF

**Figure 1:**
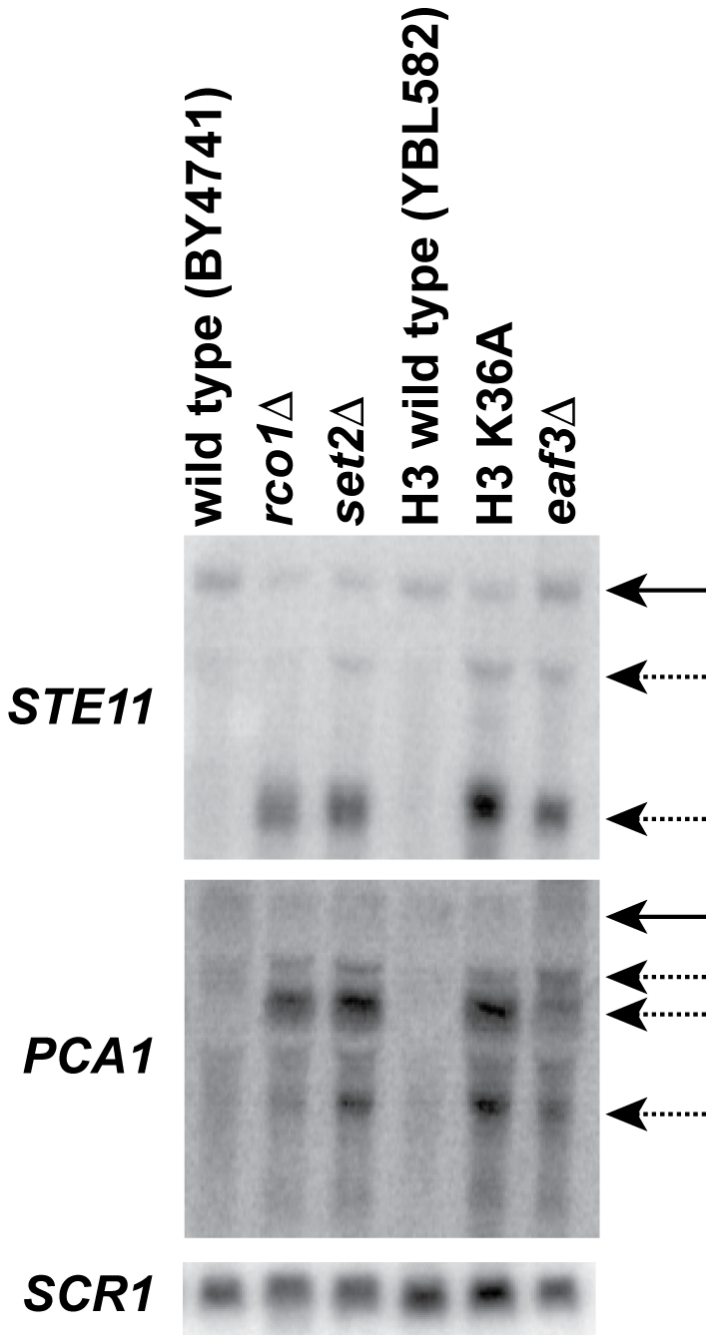
Variable transcript levels in mutants of proteins involved in the Set2-Rpd3S pathway. (A) Northern blot of RNA extracted from wild type or indicated mutant strains was probed for the 3′ regions of *STE11*, *PCA1* or for *SCR1* (loading control). Solid arrows denote full-length transcript, while dashed arrows indicate cryptic transcripts. Blot shown is representative of three biological repeats.

In addition to the full-length transcript, mutants in components of the Set2-Rpd3S pathway have cryptic internally initiated transcripts [5], [6], [7]. While cryptic transcripts originate from the same sites, they show different levels of transcript between different mutants or under different growth conditions (Figure 1, [13]). These data suggest that cryptic promoters are regulated independently of the full-length promoter, and that the factors involved in transcription activation differ from one cryptic promoter to the next.

**Figure 2:**
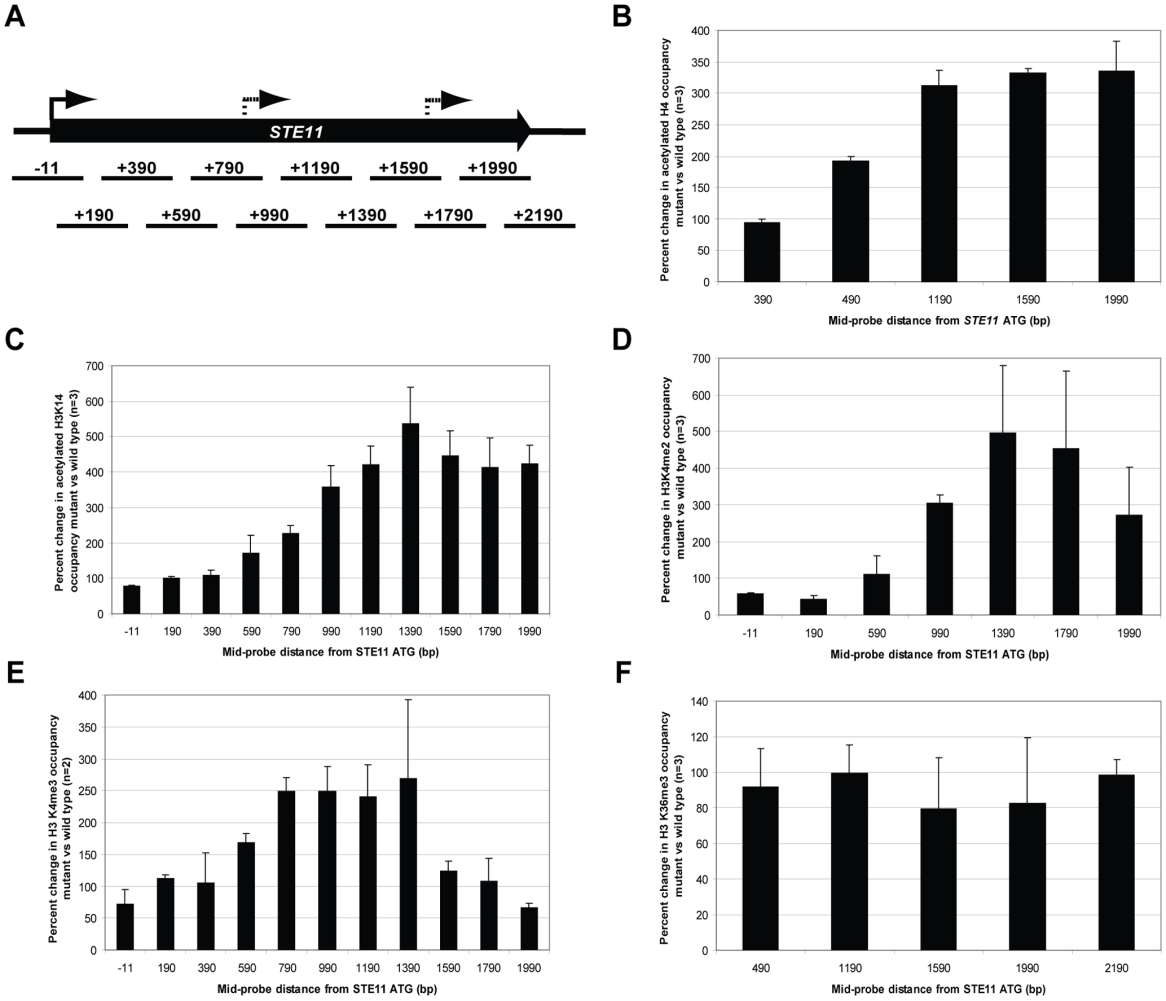
Mutants of the Rpd3S subunit, Rco1, show an increase in promoter-associated histone modifications in the ORF of the *STE11* gene. (A) Amplicons used for quantitative PCR are indicated to scale below a map of the *STE11* ORF. Numbers indicate the distance of the mid-point of each amplicon from the start site for translation of the full-length transcript (solid arrows). Cryptic promoters were mapped by 5′-RACE (dashed arrows). (B–F) ChIP using antibodies for various histone modifications was followed by quantitative PCR. All graphs represent the percent change in occupancy of the histone modification in *rco1*Δ versus wild type (BY4741) strains. Error bars represent standard deviation of three biological repeats, except (E), which is two biological repeats. Antibodies used for ChIP assays were: (B) Pan-acetylated histone H4; (C) Acetylated histone H3, lysine 14; (D) di-methylated histone H3, lysine 4; (E) tri-methylated histone H3, lysine 4; (F) tri-methylated histone H3, lysine 36.

The location of histone modifications associated with gene promoters is tightly regulated. Specifically, acetylated histone H3 (AcH3) and H4 (AcH4), and tri-methylated histone H3, lysine four (H3K4me3), are associated with the promoter and 5′ ORF region of actively transcribed genes (Reviewed in [4]). We wanted to determine what defines a cryptic promoter. Do these promoters show characteristics similar to canonical promoters? In order to address this question, we used a mutant of a subunit unique to the Rpd3S histone deacetylase complex, *rco1*Δ, which does not have HDAC function [5], [11]. Previous studies have shown that about 30% of genes in *rco1*Δ mutants have hyperacetylated ORFs [11], [12]. We chose one of these genes, *STE11*, which has two cryptic transcripts (Figure 1, top panel, lane 2). The location of the start site for each cryptic transcript was mapped by 5′-RACE (data not shown). We performed chromatin immunoprecipitation (ChIP), to determine which promoter-associated modifications were present in the gene ORF in *rco1*Δ mutants using primers tiling the *STE11* locus (Figure 2B–F). Not only was acetylated H4 increased across the ORF as previously described [11], [12], but Acetylated H3, lysine 14 (H3AcK14), dimethylated H3 lysine 4 (H3K4me2), and trimethylated H3 lysine 4 (H3K4me3) were also increased in *rco1*Δ mutants when compared to the wild type strain (Figure 1B–E). There was no change in trimethylated H3 lysine 36 (H3K36me3), which was expected since Rpd3S HDAC activity is downstream of co-transcriptional methylation activity by the Set2 histone methyltransferase [5], [6], [7], [11]. Thus, loss of functional Rpd3S results in the appearance of promoter-associated histone modifications in the ORF of the *STE11* gene. These data show that the region surrounding the transcription start site for each cryptic transcript resembles that of a canonical promoter.

### Deletion of the bromodomain-containing protein, Rsc1, partially suppresses the small *STE11* cryptic transcript

Bromodomain-containing proteins are important for recruitment of chromatin remodeling complexes to acetylated histones (reviewed in [1], [2], [14]). Subunits of the RSC complex contain multiple bromodomains, which recognize acetyl lysine residues on histones and other proteins. *In vitro*, purified RSC complex was shown to stimulate Pol II transcription through a nucleosome template; an event that was enhanced by NuA4 or SAGA-mediated histone acetylation [29]. *In vivo*, RSC activity has been implicated in nucleosome repositioning and maintenance of the nucleosome free region (NFR) at Pol II-transcribed promoters [27], [28]. We wanted to determine if RSC activity was important for the formation of cryptic promoters in gene ORFs that showed the hyperacetylation phenotype in Rpd3S mutants. We deleted Rsc1 and Rsc2, which are present in two distinct RSC subcomplexes [44], and show a very similar genome-wide occupancy profile [45]. Both proteins contain tandem bromodomains that are essential for RSC function, but not complex assembly [44]. Deletion of these proteins in combination is lethal, but deleting *RSC1* or *RSC2* individually results in cells that are viable, but show growth defects due to the loss of transcription at sporulation-specific genes [44], [46], [47].

**Figure 3:**
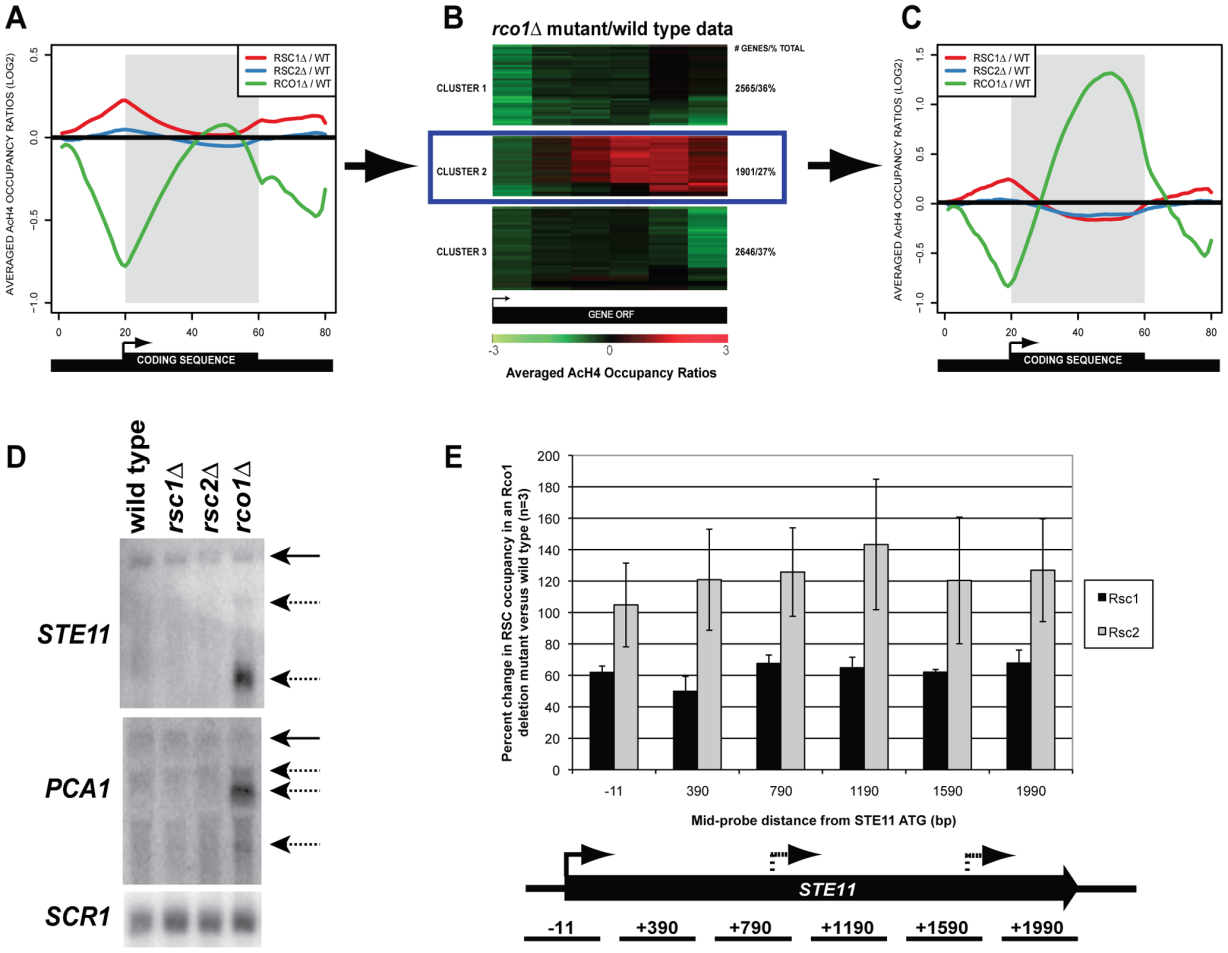
Genome-wide occupancy of acetylated H4 in *rco1*Δ, *rsc1*Δ, and *rsc2*Δ mutant strains. Indicated mutants were subjected to ChIP with an antibody directed against acetylated H4 followed by microarray analysis (n = 3) using the Agilent yeast 4x44K platform (Agilent Technologies, Santa Clara, CA). (A) Average gene analysis of log_2_ ratio for the enrichment of AcH4 in mutant versus the wild type strain (Y-axis). X-axis indicates the number of bins used for average gene analysis. The shaded grey area represents any given gene ORF. (B) ORFs from the dataset comparing enrichment of AcH4 in *rco1*Δ mutants versus wild type were divided into 6 bins [51], followed by K-means clustering using MeV [57]. The ORFs represented by Cluster 2 show an increase in AcH4 across the ORF in *rco1*Δ mutants. The scale at the bottom of the figure represents the log_2_ ratio for the enrichment of AcH4 in *rco1*Δ mutant versus the wild type ranging from −3 to 3. (C) Average gene analysis of log_2_ ratio for the enrichment of AcH4 in mutant versus the wild type strain for gene cluster 2 (genes with enrichment of AcH4 in the ORF). (D) Northern blot of RNA extracted from wild type (BY4741) or indicated mutant strains was probed for the 3′ regions of *STE11*, *PCA1* or for *SCR1* (loading control). Solid arrows denote full-length transcript, while dashed arrows indicate cryptic transcripts. Blot shown is representative of three biological repeats. (E) ChIP followed by qPCR using primers corresponding to the *STE11* ORF was performed with antibodies against c-myc-tagged Rsc1 or Rsc2. The X-axis indicates the mid-position of each probe set used in the PCR (base pairs). The Y-axis represents percent change of c-myc IP/input for *rco1*Δ mutants compared to IP/input for the wild type strain. Error bars represent standard deviation of three biological repeats.

We first examined the genome-wide effect of deleting *RSC1* or *RSC2* on acetylated H4 at genes that show hyperacetylation in *rco1*Δ mutants (Figure 3). ChIP samples were amplified using a double T7 linear amplification protocol [48], [49], [50], followed by hybridization to yeast high-resolution tiling microarrays. The log_2_ ratios of immunoprecipitated (IP) AcH4 versus input were subjected to a modified average gene analysis [51], which allowed us to examine the average AcH4 signal genome-wide at any given gene and surrounding intergenic region (Figure 3A). Using the dataset comparing enrichment of AcH4 in *rco1*Δ mutants versus wild type, we identified genes that grouped into three clusters based on their enrichment patterns (Figure 3B).

Earlier work from our group showed that the cluster of hyperacetylated ORFs (Cluster 2) was composed mainly of longer, less frequently transcribed genes [12]. These genes do not completely overlap with those that receive the highest levels of H3K36me3, nor do they all produce cryptic transcripts, although all genes that produce cryptic transcripts fall within Cluster 2 (data not shown) [12], [51]. Since we were specifically interested in cryptic internal initiation of transcription, we focused on Cluster 2 and filtered the dataset for genes with an ORF that was represented by two or more probes. As previously demonstrated, *rco1*Δ mutants showed an increase in acetylated H4 across a gene ORF when compared to the wild type strain (Figure 3C, [11], [12]). When the RSC complex mutants were compared to wild type, *rsc2*Δ showed no change, while the *rsc1*Δ strain showed an increase in AcH4 at the promoter region. Therefore, the RSC1 complex suppresses histone acetylation at yeast promoters. Neither the *rsc1*Δ nor the *rsc2*Δ strain had cryptic transcripts at *STE11* or *PCA1* (Figure 3D), which was consistent with the lack of ORF hyperacetylation in either of these mutants.

We wanted to know if the occupancy profiles of either Rsc1 or Rsc2 changed in *rco1*Δ mutants. Specifically, ChIP was performed using myc-tagged Rsc1 and Rsc2 in wild type [45] and *rco1*Δ strains, followed by PCR with primers spanning the *STE11* ORF (Figure 3E). There was no significant change in Rsc2 occupancy; however, Rsc1 occupancy was decreased by approximately forty percent in the *rco1*Δ mutant compared to the wild type strain. We do not see a direct association of the Rpd3S and RSC1 complexes by mass spectrometry (data not shown). Therefore, given that Rsc1 played a role in repression of acetylation at promoter regions in a subset of genes (Figure 3C), it is possible that retention of the RSC1 complex at certain ORFs is related to the histone deacetylation activity of Rpd3S. We attempted to examine this possibility through determination of genome-wide occupancy of Rsc1 and Rsc2 in *rco1*Δ mutants, but the data was inconclusive due to inconsistent results between biological replicates (data not shown).

**Figure 4:**
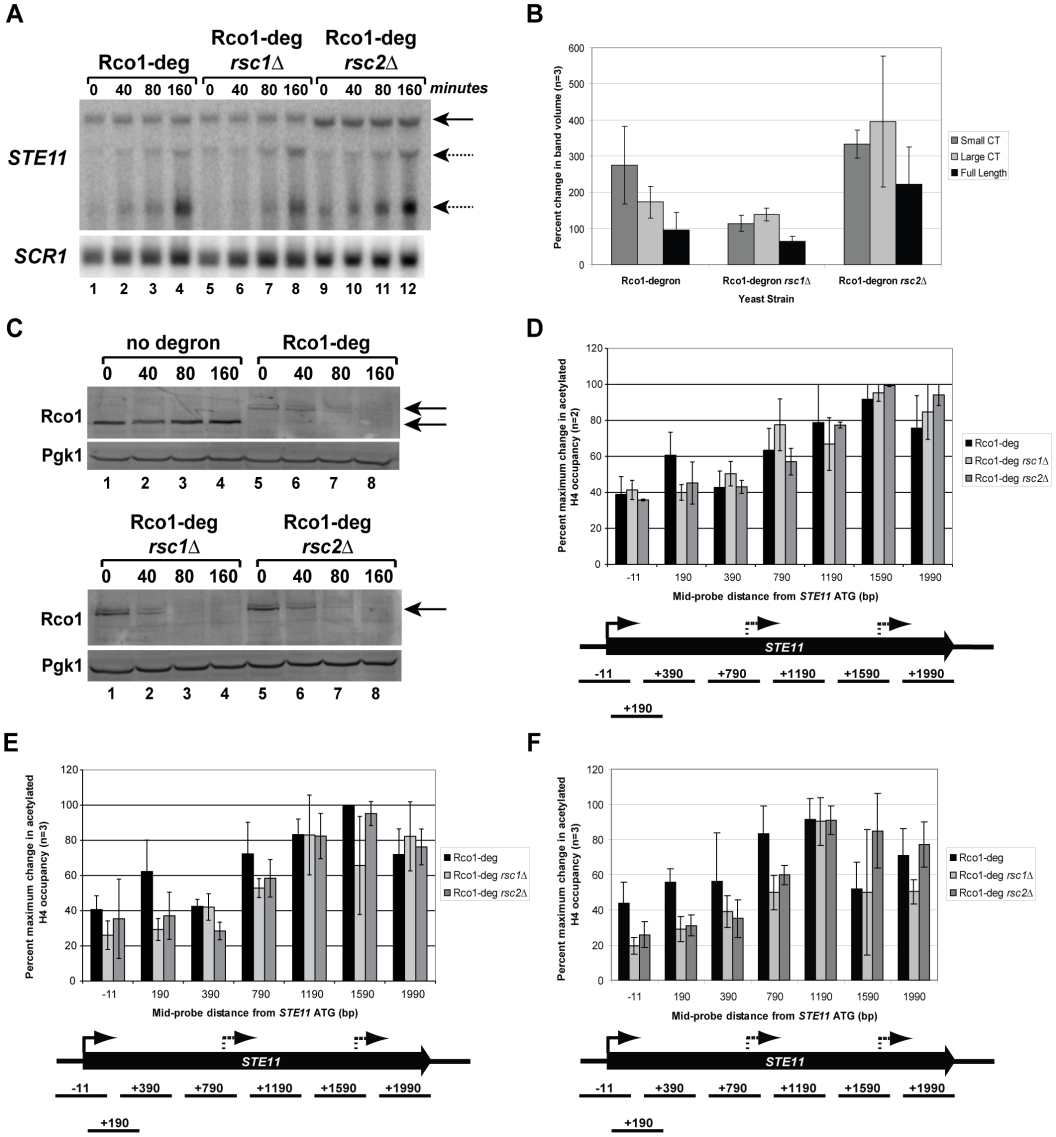
Deletion of *RSC1* in an Rco1-degron background results in a partial suppression of the small *STE11* cryptic transcript. (A) Northern blot of RNA extracted from indicated degron strains at 0, 40, 80, and 160 minutes post-degron induction. Blots were probed with amplicons from the 3′ region of *STE11* or from *SCR1* (loading control). Cryptic transcripts are indicated with dashed arrow and full-length transcripts by solid arrow. Blots are representative of 3 biological repeats. (B) Densitometry analysis of bands from northern blots in (A). Data is shown as percent change in band volume at the 160 minute time point compared to time zero (Y-axis) for each transcript in each strain (X-axis). Image Quant (GE Biosciences) was used to quantitate band density on the northern blot. All *STE11* transcripts were normalized to the loading control, *SCR1*. Error bars represent the average deviation of three biological repeats. (C) Western blot of protein extracted from degron strains at indicated time post degron induction. Tagged Rco1 protein was detected with anti-FLAG antibody. The same blots were also probed with anti-Pgk1 as a loading control. Blots are representative of 3 biological repeats. (D–F) ChIP followed by qPCR using primers corresponding to the *STE11* ORF was performed with antibodies against acetylated H4. The X-axis indicates the mid-position of each probe set used in the PCR (base pairs). The Y-axis represents percent maximum change of the ChIP product at 40 minutes (D), 80 minutes (E), or 160 minutes (F) versus 0 minutes post degron induction for each strain. Error bars represent standard deviation of three biological repeats, except (D), which is two biological repeats.

If the RSC complex is involved in nucleosome remodeling at cryptic promoters, then disruption of RSC subunits in an *rco1*Δ background should suppress cryptic transcription. Due to difficulties with making either *rsc1*Δ*rco1*Δ, or *rsc2*Δ*rco1*Δ double mutants, we created an Rco1-degron strain (Rco1-deg), using the system described in Kanemaki *et al*. [52]. The C-terminus of the Rco1 protein was tagged with a FLAG tag for detection by western blot (see materials and methods section). RNA and protein samples were extracted at 0, 40, 80, and 160 minutes following induction of Rco1 protein degradation (Figure 4A, B). Cryptic transcription was visualized by northern blot at the *STE11* gene beginning at 40 minutes post-induction (Figure 4A, Lanes 1–4). Rco1 protein levels did not change in a control with FLAG-tagged Rco1 protein that lacked the degron tag in the degron strain background (Figure 4C, upper panel, lanes 1–4). The Rco1-deg strain had no visible Rco1 protein after 80 minutes (Figure 4C, upper panel, lanes 5–8), which was consistent with the appearance of cryptic transcripts (Figure 4A, lanes 1–4).

We examined the effects of RSC on cryptic transcript formation by deleting either Rsc1 or Rsc2 in the Rco1-degron background. In both of these deletion strains, Rco1 protein was no longer visible after 80 minutes (Figure 4C, bottom panel, lanes 1–8). When *STE11* transcript levels were evaluated by northern blot, however, formation of the small cryptic transcript was delayed in the *rsc1*Δ Rco1-deg strain compared to either Rco1-deg, or *rsc2*Δ Rco1-deg strains (Figure 4A, compare lanes 5–8 to lanes 1–4 and 9–12). Densitometry of northern blots from three biological repeats of this experiment showed that formation of the small cryptic transcript was suppressed by approximately 50% in the *rsc1*Δ Rco1-deg strain, compared to Rco1-deg alone (Figure 4B). Formation of the large cryptic transcript in the *rsc1*Δ Rco1-deg strain was comparable to Rco1-degron alone (Figure 4B). Therefore the RSC1 complex, and not the RSC2 complex suppresses formation of the small cryptic transcript at the *STE11* gene in Rpd3S mutants. This finding also indicates that there is differential regulation of each cryptic promoter as the large cryptic promoter was not sensitive to *RSC1* deletion.

We next used ChIP to assess the status of acetylated H4 occupancy at the *STE11* locus at 40, 80, and 160 minutes compared to 0 minutes post-degron induction (Figure 4D–F). Maximum acetylated H4 occupancy occurred in the ORF, rather than the promoter region in all three strains at all three time points examined (Figure 4D), despite the fact that there was a difference in the intensity of the small cryptic transcript in the *rsc1*Δ Rco1-deg strain at 40 minutes (Figure 1A). Therefore differential regulation of cryptic promoters at *STE11* by the RSC1 complex is determined by events downstream of ORF acetylation.

### Deletion of the bromodomain-containing protein, Bdf1, completely suppresses the small *STE11* cryptic transcript

Bdf1, like Rsc1, is a tandem bromodomain-containing protein that is important for recruitment and retention of TFIID at TATA-less promoters [32], [38], [41], [43]. In yeast, Bdf1 serves as the bromodomain-containing portion of Taf1 [30], [31], [32], and is important for recruitment of TFIID to TATA-less promoters [43]. Since Bdf1-dependent recruitment of TFIID plays an early role in transcription activation, we examined the role of this bromodomain-containing protein in cryptic transcript formation.

**Figure 5:**
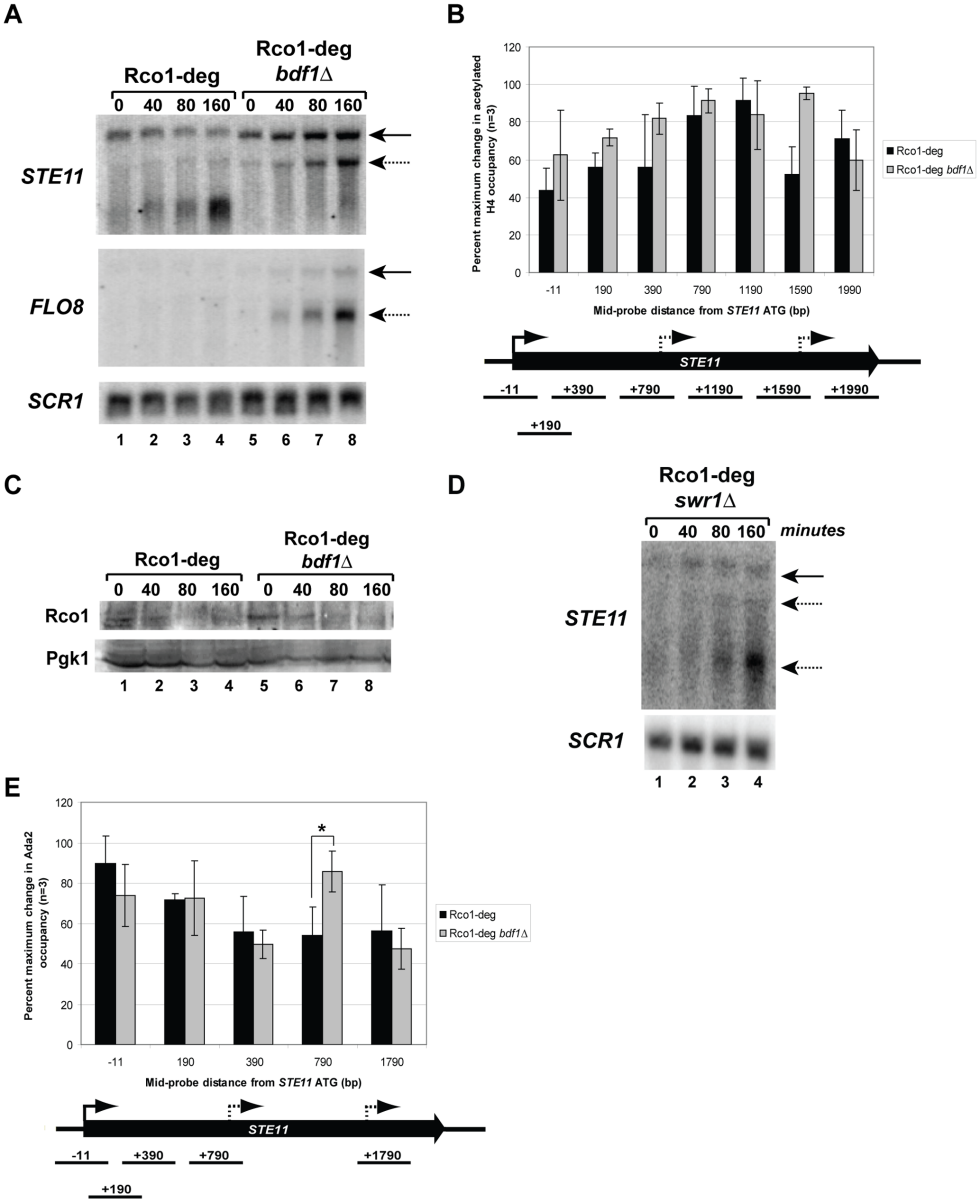
Deletion of *BDF1* in an Rco1-degron strain suppresses the small STE11 cryptic transcript. (A) Northern blot of RNA extracted from indicated degron strains at 0, 40, 80, and 160 minutes post-degron induction. Blots were probed with amplicons from the 3′ region of *STE11*, *FLO8* or *SCR1* (loading control). Cryptic transcripts are indicated with dashed arrow and full-length transcripts by solid arrow. (B) Western blot of protein extracted from degron strains at indicated time post degron induction. Rco1 protein was detected with anti-FLAG antibody. The same blots were also probed with anti-Pgk1 as a loading control. (C) ChIP followed by qPCR using primers corresponding to the *STE11* ORF was performed with antibody against acetylated H4. The X-axis indicates the mid-position of each probe used in the PCR (base pairs). The Y-axis represents percent maximum change of the ChIP product at 160 minutes versus 0 minutes post degron induction for each strain. Error bars indicate standard deviation (n = 3). (D) Northern blot of RNA extracted from *swr1*Δ, Rco1 degron strain at 0, 40, 80, and 160 minutes post degron induction. Blots were probed with amplicons from the 3′ region of *STE11* or *SCR1* (loading control). Cryptic transcripts are indicated with dashed arrow and full-length transcripts by solid arrow. (E) ChIP followed by qPCR using primers corresponding to the *STE11* ORF was performed with antibody against the SAGA subunit, Ada2. The X-axis indicates the mid-position of each probe used in the PCR (base pairs). The Y-axis represents percent maximum change of the ChIP product at 160 minutes versus 0 minutes post degron induction for each strain. Error bars represent average deviation (n = 3). The P-value of the points at 790 bp equals 0.05 as determined by a T-test (*).

We deleted Bdf1 from the Rco1-degron strain and determined the formation of cryptic transcripts over time at the *STE11* ORF (Figure 5A, top panel). Western blots showed that Rco1 degradation in the *bdf1*Δ Rco1-deg strain was comparable to that in Rco1-deg alone (Figure 5C, compare lanes 1–4 and 5–8). When we examined transcript formation by northern blot, however, we were surprised to find that the small cryptic transcript was completely suppressed in the *bdf1*Δ Rco1-deg strain (Figure 5A, top panel, compare lanes 4 and 8). Also interesting, was the fact that both the large cryptic transcript and the full-length transcript increased in intensity. These results indicate that dependence on co-activators for transcription activation varies from one cryptic promoter to the next. We also looked at cryptic transcript formation at the *FLO8* locus, which has a single cryptic transcript. Compared to the Rco1-deg strain alone, the *bdf1*Δ Rco1-deg strain showed a dramatic increase in both the full-length and cryptic transcript at the *FLO8* gene (Figure 5A, middle panel). When AcH4 occupancy at the *STE11* locus was determined by ChIP, both Rco1-deg and the *bdf1*Δ Rco1-deg strain had maximum occupancy of this modification in the gene ORF (Figure 5B). Therefore, like RSC, Bdf1 affects cryptic promoter activity downstream of histone acetylation.

Bdf1 also interacts with the SWR1 complex, which is responsible for deposition of the H2A.Z histone variant [33], [34], [35]. We wanted to determine if the suppression of the small cryptic transcript in the *bdf1*Δ Rco1-deg strain was related to Bdf1 recruitment of SWR1. Deletion of the catalytic subunit, Swr1, from the Rco1-degron strain had no effect on the formation of cryptic transcripts at the *STE11* ORF (Figure 5D, Lanes 2–4). Thus, the function of Bdf1 at cryptic promoters is probably independent of its role in the recruitment of the SWR1 complex. An interesting future experiment would be to compare the genome-wide occupancy of Bdf1 and other components of the TFIID complex to the locations of cryptic transcription in *rco1*Δ mutants.

Genes that rely on Bdf1 for TFIID recruitment are generally not associated with SAGA [41], [43]. In fact, Bdf1 has been linked to repression of SAGA-dependent genes [32], [39], [40], [41]. Since there was a loss of the small cryptic transcript at *STE11*, but an increase in the intensity of the large cryptic and full-length transcripts (Figure 5A), we compared SAGA occupancy at this gene between the Rco1-degron and *bdf1*Δ Rco1-degron strains. ChIP was performed with an antibody directed against the Ada2 subunit of SAGA, followed by qPCR with primers directed against the *STE11* locus (Figure 5E). In the Rco1-degron strain (black bars), maximal Ada2 occupancy occurs at the full-length *STE11* promoter region. Occupancy at the large (+790) and small (+1790) cryptic promoter regions was comparable to that of a probe located in a region that does not contain a cryptic promoter (+390). This high baseline of Ada2 occupancy is likely to contribute to the increased levels of acetylation across the ORF as shown in Figure 4. In the absence of Bdf1, however (grey bars), maximal occupancy at the large promoter (+790) increased to a level comparable to the full-length promoter (−11). There was no significant change at the small cryptic promoter in the presence or absence of Bdf1 (+1790). These results, along with the northern blot data (Figure 5A), suggest that both the full-length and large cryptic promoters are SAGA-dependent, while the small cryptic promoter is SAGA-independent for expression.

Overall, cryptic promoters are independently regulated by a variety of co-activators. In this sense, they resemble canonical gene promoters, which may explain why the location of these cryptic transcription start sites does not vary like the levels of expression.

## Discussion

### Histone modifications alone do not dictate the location of cryptic transcription initiation in strains lacking functional Rpd3S

Disruption of Rpd3S function resulted in hyperacetylation at about 30% of yeast genes genome-wide. Locally, we showed that several promoter-associated histone modifications increased across the *STE11* ORF, including AcH4, AcH3K14, and H3K4me3. These histone modifications gave the entire *STE11* ORF promoter-like characteristics. Yet, cryptic transcript initiation did not randomly occur throughout the ORF; instead it initiated from two distinct positions that could be mapped by 5′-RACE (data not shown). Thus, histone modifications alone do not dictate the position of the cryptic transcription start site. Specific locations for cryptic initiation could be due to a number of additional factors that affect transcription from canonical promoters including the availability of binding sites for co-transcriptional activators, and the presence or absence of an exposed DNA element such as a TATA box.

### RSC1 and RSC2 complexes affect cryptic transcription differently in a mutant Rpd3S background

Rsc1 and Rsc2 are present in two distinct complexes [44] with a similar genome-wide profile [45]. Genetic evidence suggests that these two complexes function differently during sporulation [46], [47]. One study demonstrated that *rsc1*Δ mutants produce aberrant asci which could not be rescued by *RSC2* overexpression [47].

Our genome-wide data shows that deletion of *RSC1* alone results in an increase in 5′ histone H4 acetylation compared to wild type that is not seen in *RSC2* mutants. At the *STE11* locus, deletion of *RSC1* in an Rco1-degron background resulted in a partial suppression of the small cryptic transcript. When *RSC2* was deleted in the Rco1-degron strain, the intensity of the small cryptic transcript actually increased. These data provide further evidence that the RSC1 and RSC2 complexes have different functions. It also shows that the cryptic and full-length promoters are differentially regulated.

At Pol II promoters, RSC activity generally involves single nucleosome events that help to form and maintain the NFR [27], [28]. These data were generated from studies using a temperature sensitive mutant of the Sth1 catalytic subunit, which is common to both the RSC1 and RSC2 complexes [44]. We found that only the RSC1 complex affects genome-wide 5′ acetylated H4 levels, and that the small *STE11* cryptic transcript is sensitive to *RSC1* deletion. It is possible that in the absence of the RSC1 complex, certain promoters are not able to maintain an NFR and therefore have greater histone density at the promoter region. Future studies could examine the role of RSC1 complex versus RSC2 complex in the establishment and maintenance of Pol II promoter NFRs.

### The differential effect of Bdf1 on cryptic promoter formation suggests that underlying DNA sequence elements are important for cryptic promoter formation

The differential role that the RSC1 and RSC2 complexes play in transcription from cryptic promoters indicates that co-activator dependence varies just as it does with canonical promoters. Our data showing that deletion of *BDF1* in the Rco1-degron background completely suppresses only the small cryptic transcript further confirms this finding. It also suggests that DNA elements are important for initiation of cryptic transcription since Bdf1 is known to affect a very specific subset of promoters. At the *PHO5* promoter, when the TATA box was obstructed by a re-positioned nucleosome, gene expression became entirely dependent on the presence of Bdf1 [43]. TFIID dependence on Bdf1 for promoter recruitment is common at housekeeping genes that generally have TATA-less promoters. While there are no TATA boxes fitting the criteria TATA(A/T)A(A/T)(A/G) [42] upstream of *STE11* full-length or cryptic promoters, we did note the same increase of the *FLO8* full-length and cryptic transcripts, both of which are known have TATA boxes [13]. Therefore, the dependence of the small cryptic transcript on Bdf1 could stem from the fact that it is a TATA-less promoter, or that a degenerate TATA box is obstructed by a repositioned nucleosome. An interesting future experiment would be to compare the genome-wide occupancy of Bdf1 and other components of the TFIID complex to the locations of cryptic transcription and known TATA-containing promoters in *rco1*Δ mutants.

In contrast to the housekeeping genes, stress-induced genes characteristically have promoters that do not require Bdf1, have a TATA box, and show a strong correlation with SAGA activity [32], [39], [40], [41]. We know from northern blots that prior to degron induction, the *bdf1*Δ Rco1-deg strain has full-length *STE11* transcript, so the full-length promoter is not dependent on Bdf1 for TFIID recruitment. Expression of *STE11* is inhibited 2.2 fold compared to wild type cells when the catalytic histone acetyltransferase subunit of SAGA, Gcn5, is deleted [53], suggesting that *STE11* expression is SAGA-dependent. Also, the full-length and large cryptic transcripts increase in intensity following deletion of Bdf1, which is thought to inhibit SAGA-dominated promoters [41]. Finally, Ada2 ChIP data indicated that SAGA was present at the full-length STE11 promoter, and that its maximum occupancy increased at the large cryptic promoter when Bdf1 was deleted in the Rco1-degron background. Taken together, these findings are consistent with a role for SAGA at the full-length and large cryptic *STE11* promoters. In contrast, the small cryptic promoter lacked SAGA occupancy and was completely dependent on the presence of Bdf1 for transcription activation.

Overall, cryptic transcription in yeast mutant Rpd3S strains provides an excellent system in which to study the role of various regulatory factors in preinitiation complex formation. We showed that bromodomain-containing proteins are important for transcription activation at acetylated promoters, and that cryptic promoters are regulated as independent units that follow a process of activation similar to canonical promoters.

## Methods

### Yeast Strains

See Table S1 for a list of strains used in this study.

### Antibodies

ChIP assays were performed using the following antibodies: Anti-hyperacetylated Histone H4 (Penta), Upstate #06-866; Anti-c-myc, Roche Applied Science #11667149001; Histone H3 (tri methyl K36), Abcam #ab9050; Anti-dimethyl-Histone H3 (lys4), Millipore #07-030; Anti-acetyl-Histone H3 (lys14), Millipore #07-353; Anti-Ada2 [54]; and Histone H3 antibody, Abcam #ab1791. Western blots were performed with Anti-FLAG M2 monoclonal antibody, Sigma #A8592; and Phosphoglycerate Kinase Monoclonal Antibody, Invitrogen #459250.

### Chromatin Immunoprecipitation Assays

Chromatin immunoprecipitation (ChIP) assays were performed as previously described [12], except 50 uL of protein G Dynabeads (Invitrogen) were used for the immunoprecipitation. Yeast strains were grown in YPD except for strains containing the degron-tagged Rco1. Degron strains were grown as described in [52].

### Northern Blot Analysis

Total RNA was extracted as described in [12]. Northern blotting and hybridization was performed as described in [5]. Probes against *STE11*, *PCA1*, *FLO8* and *SCR1* were generated using primers described in Table S2. Densitometry analysis was performed using ImageQuant TL v2003.02 software for the Typhoon phosphorimager (GE Healthcare).

### Quantitative PCR

DNA from ChIP assays was amplified with primers covering the *STE11* ORF (Table S2). Real time PCR was performed on a Biorad iCycler using FastStart SYBR Green Master Mix (Roche). Total nanograms of input and immunoprecipitated samples were determined by comparison to a standard yeast genomic DNA curve amplified with the same primer sets. Unless otherwise indicated, all experiments represent the average of three separate biological repeats with two technical replicates each.

### T7 Linear Amplification

The double T7 linear amplification protocol was adapted from [48], [49], [50]. For the first round reaction, up to 500 ng of ChIP or input DNA was treated with 2.5 U CIP enzyme (NEB) for 1 hour at 37°C, followed by phenol:chloroform extraction. Fifty ng of CIP-treated template was incubated with 20 U TdT (NEB) for 20 minutes at 37°C, and the reaction product was isolated using a MinElute Reaction Cleanup Kit (Qiagen). The fill reaction was then performed using an anchored T7-(dA)_18_ oligo (T7-(dA)18, Table S2) and 5 U Exo- Klenow (NEB) for 4 hours at 37°C, followed by phenol:chloroform extraction. *In vitro* transcription was then performed using the Ampliscribe T7 kit (Epicentre Biotechnologies). Amplified RNA (aRNA) was purified using the RNeasy Mini Kit (Qiagen) and quantified on a Nanodrop 2000 Spectrophotometer (Thermo Scientific). For the second round amplification, 50–150 ng of aRNA was reverse transcribed using Superscript III reverse transcriptase (Invitrogen), followed by reaction cleanup with the MinElute Reaction Cleanup Kit (Qiagen). A fill reaction followed by *in vitro* transcription was performed as described above, except amino allyl-UTP (Ambion) was added to the reaction. Final reaction cleanup was performed with the RNeasy Mini Kit (Qiagen). For the labeling reactions, 4–6 ug amino allyl-incorporated aRNA in a 5 uL volume of 0.1 M Carbonate Buffer, pH 8.7, was mixed with 5 uL (0.01 nmol) monofunctional NHS-ester Cy3 or Cy5 dye in DMSO (Sigma) and incubated at 22°C for 2 hours. Reactions were quenched with 5 uL 4 M hydroxylamine at 22°C for 15 minutes, cleaned with an RNeasy MinElute Cleanup Kit (Qiagen), and the efficiency of dye incorporation measured using the Nanodrop 2000 spectrophotometer (Thermo Scientific). Samples with a label incorporation efficiency of 2–4% were used for microarray hybridization.

### Microarray Analysis

Input was labeled with Cy3 dye, while immunoprecipitated samples were labeled with Cy5 dye. Samples were combined 1∶1 based on quantity (ng). Before hybridization, labeled aRNA samples were fragmented (Fragmentation Reagent Kit, Ambion) according to manufacturer's instructions. The hybridization mixture was set up for the Agilent yeast 4x44K platform (Agilent Technologies) according to manufacturer's instructions with the addition of 20 ug of T7 blocking oligo (Table S2). Microarray hybridization and washing was conducted according to manufacturer's instructions. Scanning was performed on the Agilent DNA Microarray Scanner (Agilent Technologies, Model#G2505B), and features extracted using Feature Extraction software (Agilent Technologies). All samples are representative of three biological repeats. Microarray data are MIAME compliant. Raw data has been deposited in a MIAME compliant database accessible through NCBI GEO [55] (GSE17521).

### Averaged Gene and Cluster Analyses

Final datasets from ChIP-chip experiments were pipelined into a modified average gene analysis based on the frame work originated by the Young laboratory [51], as described in [11]. Agilent Feature Extraction Software (v 10.5.1.1) was used to quantify images. Data was read into R and normalized within arrays using median normalization and between arrays using Aquantile normalization from the Limma package [56]. Clustering was performed as follows: ORFs from the dataset comparing enrichment of AcH4 in *rco1*Δ mutants versus wild type were divided into 6 bins [51], followed by K-means clustering using MeV [57], which is part of the Tm4 Microarray Software Suite [58].

## Supporting Information

Table S1 List of yeast strains used in this study.
(0.14 MB DOCX)

Table S2 List of primers used in this study.
(0.15 MB DOCX)
