# Supplementary material for: Features of Cryptic Promoters and Their Varied Reliance on Bromodomain-Containing Factors
Source: PLoS One. 2010 Sep 23;5(9):e12927. doi: 10.1371/journal.pone.0012927 (PMC2944879; doi:10.1371/journal.pone.0012927)
Supplement: Table S1 — List of yeast strains used in this study. (0.14 MB DOCX) [file pone.0012927.s001.docx]

**Table S1. List of yeast strains used in this study.**

| **Name** | **Genotype** | **Source** |
| --- | --- | --- |
| BY4741 (wild type) | MAT**a** *his3∆1 leu2∆0 met15∆0 ura3∆0* | Open Biosystems |
| RCO1∆ | MAT**a** *his3∆1 leu2∆0 met15∆0 ura3∆0 rco1Δ::KANMX6* | Open Biosystems |
| SET2Δ | MAT**a** *his3∆1 leu2∆0 met15∆0 ura3∆0 set2Δ::KANMX6* | Open Biosystems |
| YBL575 | MAT**a**, *leu2∆1, his3∆200, ura3-52, trp1∆63, lys2-128δ, (hht1-hhf1)∆LEU2 (hht2-hhf2)∆::HIS3 Ty912∆35-lacZ::his4* [pDM18-hht2-K36A-TRP1] | 1 |
| YBL582 | MAT**a**, *leu2∆1, his3∆200, ura3-52, trp1∆63, lys2-128δ, (hht1-hhf1)∆LEU2 (hht2-hhf2)∆::HIS3 Ty912∆35-lacZ::his4* [pDM18-HHT2-HHF2-TRP1] | 1 |
| EAF3Δ | MAT**a** *his3∆1 leu2∆0 met15∆0 ura3∆0 eaf3Δ::KANMX6* | Open Biosystems |
| YBL636 | MAT**a** *ura3-52 trp1Δ63 his3Δ200 leu2::PET56 Rsc1-9myc::TRP* | 3 |
| YBL637 | MAT**a** *ura3-52 trp1Δ63 his3Δ200 leu2::PET56 Rsc2-9myc::TRP* | 3 |
| YSP32 | MAT**a** *ura3-52 trp1Δ63 his3Δ200 leu2::PET56 Rsc1-9myc::TRP rco1Δ:KANMX6* | 3, this study |
| YSP33 | MAT**a** *ura3-52 trp1Δ63 his3Δ200 leu2::PET56 Rsc2-9myc::TRP rco1Δ:KANMX6* | 3, this study |
| YSP252 | MAT**a** *ade2-1 ura3-1 his3-11,15 trp1-1 leu2-3,112 can1-100 UBR1::GAL-HA-UBR1 (HIS3) RCO1-2XFLAG::leu2* | 2, this study |
| YSP253 | MAT**a** *ade2-1 ura3-1 his3-11,15 trp1-1 leu2-3,112 can1-100 UBR1::GAL-HA-UBR1 (HIS3) rco1::CUP1-myc-rco1 (KANMX6) RCO1-2XFLAG::leu2* | 2, this study |
| YSP254 | MAT**a** *ade2-1 ura3-1 his3-11,15 trp1-1 leu2-3,112 can1-100 UBR1::GAL-HA-UBR1 (HIS3) rco1::CUP1-myc-rco1 (KANMX6) rsc1∆::TRP1 RCO1-2XFLAG::leu2* | 2, this study |
| YSP255 | MAT**a** *ade2-1 ura3-1 his3-11,15 trp1-1 leu2-3,112 can1-100 UBR1::GAL-HA-UBR1 (HIS3) rco1::CUP1-myc-rco1 (KANMX6) rsc2∆::TRP1 RCO1-2XFLAG::leu2* | 2, this study |
| YSP261 | MAT**a** *ade2-1 ura3-1 his3-11,15 trp1-1 leu2-3,112 can1-100 UBR1::GAL-HA-UBR1 (HIS3) rco1::CUP1-myc-rco1 (KANMX6) RCO1-2XFLAG::leu2 bdf1∆::TRP1* | 2, this study |
| YSP264 | MAT**a** *ade2-1 ura3-1 his3-11,15 trp1-1 leu2-3,112 can1-100 UBR1::GAL-HA-UBR1 (HIS3) rco1::CUP1-myc-rco1 (KANMX6) RCO1-2XFLAG::leu2 swr1∆::TRP1* | 2, this study |

1. Carrozza MJ, Li B, Florens L, Suganuma T, Swanson SK, et al. (2005) Histone H3

methylation by Set2 directs deacetylation of coding regions by Rpd3S to suppress

spurious intragenic transcription. Cell 123: 581-592.

2. Kanemaki M, Sanchez-Diaz A, Gambus A, Labib K (2003) Functional proteomic

identification of DNA replication proteins by induced proteolysis in vivo. Nature

423: 720-724.

3. Ng HH, Robert F, Young RA, Struhl K (2002) Genome-wide location and regulated

recruitment of the RSC nucleosome-remodeling complex. Genes Dev 16: 806-819.
