# Supplementary material for: Features of Cryptic Promoters and Their Varied Reliance on Bromodomain-Containing Factors
Source: PLoS One. 2010 Sep 23;5(9):e12927. doi: 10.1371/journal.pone.0012927 (PMC2944879; doi:10.1371/journal.pone.0012927)
Supplement: Table S2 — List of primers used in this study. (0.15 MB DOCX) [file pone.0012927.s002.docx]

**Table S2. List of primers used in this study.**

| **Name** | **Primer Sequence (5’ to 3’)** | **Used For** | **Reference** |
| --- | --- | --- | --- |
| T7-(dA)18 | GGA GGC CGG AGA ATT GTA ATA CGA CTC ACT ATA GGG AGA CGC GTG AAA AAA AAA AAA AAA AAA B | T7 Amplification | 2 |
| T7 Blocking Oligo | TTT TTT TTT TTT TTC ACG CGT CTC CC | Microarray hybridization | 2 |
| SCR1 QPCR F | AGG CTG TAA TGG CTT TCT GGT GGG A | Northern blot probe | 1 |
| SCR1 QPCR R | ATA TGT GCT ATC CCG GCC GCC TCC A | Northern blot probe | 1 |
| o-STE11-11 | GAA GGA GTT ACA TCA TGA GAA CAT TGT TAC | Northern blot probe | This Study |
| o-STE11-12 | GTG TGC ATC CAG CCA TGG ATG CTG CAG CAA | Northern blot probe | This Study |
| o-PCA1-15 | GTC ATA ATT CTC AAT CTT TGT TGC TTG GAT | Northern blot probe | This Study |
| o-PCA1-16 | CTA GCT TGG CAA CC TCA CTT CCC TCA TTG A | Northern blot probe | This Study |
| o-FLO8-13 | GAC GCT CAG AAG CAA AGA AGT TCT AAG GTA | Northern blot probe | This Study |
| o-FLO8-14 | CTC AAC ACG TGA CTT CAG CCT TCC CAA TTA |  |  |
| STE 11 I F | CTT TCG TTA TCA GGC TAG CAT | ChIP, qPCR | This Study |
| STE 11 I R | AGG TAA ATC GTT GGT CTT TTC | ChIP, qPCR | This Study |
| STE 11 II F | GAC GAA AAG ACC AAC GAT TTA | ChIP, qPCR | This Study |
| STE 11 II R | CAC CTG TTC AAT CCG TTT ATC | ChIP, qPCR | This Study |
| STE 11 III F | TTC CAG AGA GAT AAA CGG ATT | ChIP, qPCR | This Study |
| STE 11 III R | TTC ATG TGG CAA TCT TCT GAT | ChIP, qPCR | This Study |
| STE 11 IV F | GCT AAT CAG AAG ATT GCC ACA | ChIP, qPCR | This Study |
| STE 11 IV R | TGC ACT CAA CGT TCT TAG ATA | ChIP, qPCR | This Study |
| STE 11 V F | TAT CTA AGA ACG TTG AGT GCA | ChIP, qPCR | This Study |
| STE 11 V R | TCT CTC ATC GTC TTT TGC AAC | ChIP, qPCR | This Study |
| STE 11 VI F | CCG GAT ATT TTC CTC ATA CAG | ChIP, qPCR | This Study |
| STE 11 VI R | CTT CGG AAT TTT AGT TGT GTC | ChIP, qPCR | This Study |
| STE 11 VII F | CTT GAC ACA ACT AAA ATT CCG | ChIP, qPCR | This Study |
| STE 11 VII R | CAA GTA AAC ACT CCC AAA ACT | ChIP, qPCR | This Study |
| STE 11 VIII F | GCA GTT TTG GGA GTG TTT ACT | ChIP, qPCR | This Study |
| STE 11 VIII R | CAT CAA CCA TCT TTC TGT GAA | ChIP, qPCR | This Study |
| STE 11 IX F | TTC ACA GAA AGA TGG TTG ATG | ChIP, qPCR | This Study |
| STE 11 IX R | CTT CTT ATG CAA ATA CGC AAC | ChIP, qPCR | This Study |
| STE 11 X F | GTC CAT TTG AGG AAT CAC TGA | ChIP, qPCR | This Study |
| STE 11 X R | ACA TCC AGA ATA CGG AAC CTT | ChIP, qPCR | This Study |
| STE 11 XI F | AAG GTT CCG TAT TCT GGA TGT | ChIP, qPCR | This Study |
| STE 11 XI R | CCA ACT CAA ATG CCT TTC TTA | ChIP, qPCR | This Study |
| STE 11 XII F | GCT ACG TCA GAA GGA AAG AAT | ChIP, qPCR | This Study |
| STE 11 XII R | AAA CCG GTA AAT AGC AGA TAT | ChIP, qPCR | This Study |

1. Carrozza MJ, Li B, Florens L, Suganuma T, Swanson SK, et al. (2005) Histone H3

methylation by Set2 directs deacetylation of coding regions by Rpd3S to suppress

spurious intragenic transcription. Cell 123: 581-592.

2. van Bakel H, van Werven FJ, Radonjic M, Brok MO, van Leenen D, et al. (2008)

Improved genome-wide localization by ChIP-chip using double-round T7 RNA

polymerase-based amplification. Nucleic Acids Res 36: e21.
